# An exploratory assessment of icodextrin for ultrafiltration failure subtypes in peritoneal dialysis

**DOI:** 10.1038/s41598-026-56076-6

**Published:** 2026-06-06

**Authors:** Chuishun Yang, Wenjuan Lei, Xingli Cai, Jun Zhou

**Affiliations:** 1 Department of Nephrology and Rheumatology, Haikou People’s Hospital Affiliated to Xiangya School of Medicine, Haikou, China; 2 43 Renmin Road, Haidian Island, Haikou, China

**Keywords:** Ultrafiltration failure, Icodextrin, Ultrafiltration, Sodium sieving, Osmotic conductance to glucose, Diseases, Medical research, Nephrology

## Abstract

Although icodextrin was initially developed for rapid transporters (type 1 UFF), emerging evidence suggests its effects might be independent of peritoneal membrane transport characteristics. This study aimed to evaluate the suitability of 7.5% icodextrin for all PD patients with UFF. We enrolled 19 patients with UFF and 24 non-UFF PD controls. All participants underwent standard, modified, Mini-, and Double-Mini peritoneal equilibrium tests (PET). Key parameters assessed included sodium sieving (∆DNa60, ∆D/PNa60), osmotic conductance to glucose, small-pore ultrafiltration (UFSP), free water transport (FWT), and net ultrafiltration. Correlation analyses were conducted between icodextrin-induced UF and these metrics. Descriptive analysis revealed variations in icodextrin UF, ∆D/PNa60, and FWT were identified across the four UFF types. Numerically, icodextrin UF was highest in type 1 and type 2 UFF, while FWT appeared lower in type 2. Net icodextrin UF correlated positively with ∆D/PNa60, ∆DNa60, and UFSP. Our findings indicate that 7.5% icodextrin may be suitable for PD patients with type 1 and 2 UFF, but appears perhaps less effective in those with type 3 and 4 UFF.Moreover, icodextrin ultrafiltration may be predictable through UFSP measurement, offering a potential clinical tool for patient management.

## Introduction

Ultrafiltration failure (UFF) refers to the inadequate removal of excess fluid during dialysis. In peritoneal dialysis (PD), this complication is frequently observed, and its incidence tends to increase with increasing duration of treatment. UFF accounts for approximately 18% of all technique failures and the subsequent transition to hemodialysis^1^. UFF is characterized by less than 400 ml of ultrafiltration (UF) following a 4-hour exchange using 2,000 ml of a 3.86% glucose dialysis solution^2^. Following the assessment of UFF, the peritoneal membrane should be evaluated using the peritoneal equilibrium test (PET). UFF is composed of four distinct types, types 1 through 4^2^. Type 1 UFF, characterized by reduced ultrafiltration volume and rapid solute transport, can be either an intrinsic or acquired condition (such as following peritonitis or prolonged peritoneal dialysis). This condition occurs due to the dissipation of the osmotic gradient caused by glucose absorption into the bloodstream^3^. Type 2 UFF presents with a decrease in ultrafiltration volume accompanied by reduced solute transport. The primary cause of type 3 UFF is a reduction in the effective peritoneal surface area for exchange, often from fibrosis. Additionally, decreased osmotic conductance in response to glucose results in insufficient water removal through aquaporins. The reduction in osmotic conductance to glucose (OCG) is associated with a significant decrease in sodium sieving, often attributed to impaired aquaporin-1 function. Type 4 UFF results from an increased rate of bulk fluid absorption from the peritoneal cavity into the lymphatic system and surrounding tissues^3^.

Icodextrin is the only large-molecular-weight osmotic agent used in PD solutions^4^. It promotes transcapillary ultrafiltration through a mechanism similar to ‘colloid’ osmosis, primarily through small pores. Icodextrin-based solutions result in minimal solute sieving, enhancing convective transport and the clearance of small solutes. Compared with glucose, icodextrin provides a longer-lasting positive ultrafiltration effect due to its slower absorption, which is attributed to its larger molecular weight. Therefore, it is theoretically ideal for patients with poor ultrafiltration, especially for type 1 UFF patients, due to rapid glucose absorption. Consequently, it was initially intended solely for rapid transporters^3^. However, previous studies suggest that the effects of icodextrin may be largely independent of peritoneal membrane characteristics^5^. A 4-week randomized double-blind trial demonstrated that the increase in UF was most pronounced in higher transporters, although significant increases were also observed in high, high-average, and even low-average transporters^6^. Patients with average or low transport may also develop UFF, so the question of whether icodextrin could benefit them is raised. This study aimed to determine if the 7.5% icodextrin solution is suitable for all PD patients with UFF.

## Methods

### Patients

We enrolled patients with UFF and stable PD patients without UFF from our PD unit between January 2022 and January 2024.The study was approved by the Institutional Review Board of Human Research Ethics Committee of Haikou People’s Hospital Affiliated to Xiangya School of Medicine (Approval No. SC20220449). All patients used commercially available PD solutions. On average, patients had been on PD for 76 months (ranging from 2 to 166 months) prior to starting this regimen (Table 1). At the time of testing, all patients had been free from peritonitis for 4 weeks. We recorded each patient’s sex, age, PD vintage, sodium sieving, transport status, and type of UFF while using a regimen that included one icodextrin solution. All patients underwent one icodextrin exchange each day for three days, each lasting 8 to 12 h. The net UF from icodextrin was obtained as an average of the three-day UF volume.

**Table 1:** Demographic and clinical characteristics of peritoneal dialysis patients with ultrafiltration failure.

Characteristics	UFF group	NUFF group	Value
Age at start of PD(years) Gender(male/female) PD vintage (months) Etiology of ESRD [n] Glomerulonephritis Diabetic nephrology Polycystic kidney Chronic nephritic syndrome Lupus Nephritis	43.89 ± 12.18 11/8 76.21 ± 40.45 8 4 3 3 1	46.59 ± 8.58 17/7 27.92 ± 24.72 8 10 2 3 1	0.058 0.514 0.000 0.126
Type of ultrafiltration failure Type 1 Type 2 Type 3 Type 4	19 6 8 3 2		
Peritoneal transport Low Low-average High-average High	19 1 7 7 4	24 2 12 5 5	0.303

All participants provided written informed consent. All methods were carried out in accordance with the Declaration of Helsinki and relevant guidelines and regulations.

### PET

PET characteristics were evaluated using standard PET. In all patients, modified PET, Mini-PET, and Double Mini-PET were conducted biannually for routine evaluation of the peritoneal membrane. The Double Mini-PET includes two Mini-PET procedures conducted in succession: a 1.36% glucose solution is utilized for the initial test, while a 3.86% glucose solution is employed for the second. These tests provide data on small solute kinetics (D4/D0 glucose and the dialysate-to-plasma ratio of creatinine (D/PCr)), sodium sieving, OCG, UF through small pores (UFSP), and Net UF. Free water transport (FWT) was calculated from the 1-hour 3.86% glucose Mini-PET.The modified PET was performed using a 3.86% glucose dialysate, with temporal drainage at one hour and final drainage at four hours. Net UF was also measured using the modified PET.

Patients were categorized into four transport groups: D/PCr at 4 h (high transporters (HT) > 0.81), high average (HAT) 0.65–0.81, low average (LAT) 0.50–0.64, and low (LT) < 0.50), and dialysate glucose ratios at 4 and 0 h of dwell (D4/D0) were used for classification (high < 0.26, high average 0.26–0.38, low average 0.39–0.49, and low > 0.49)^7^.

### Diagnosis of the causes of UUF

**Fig. 1:**
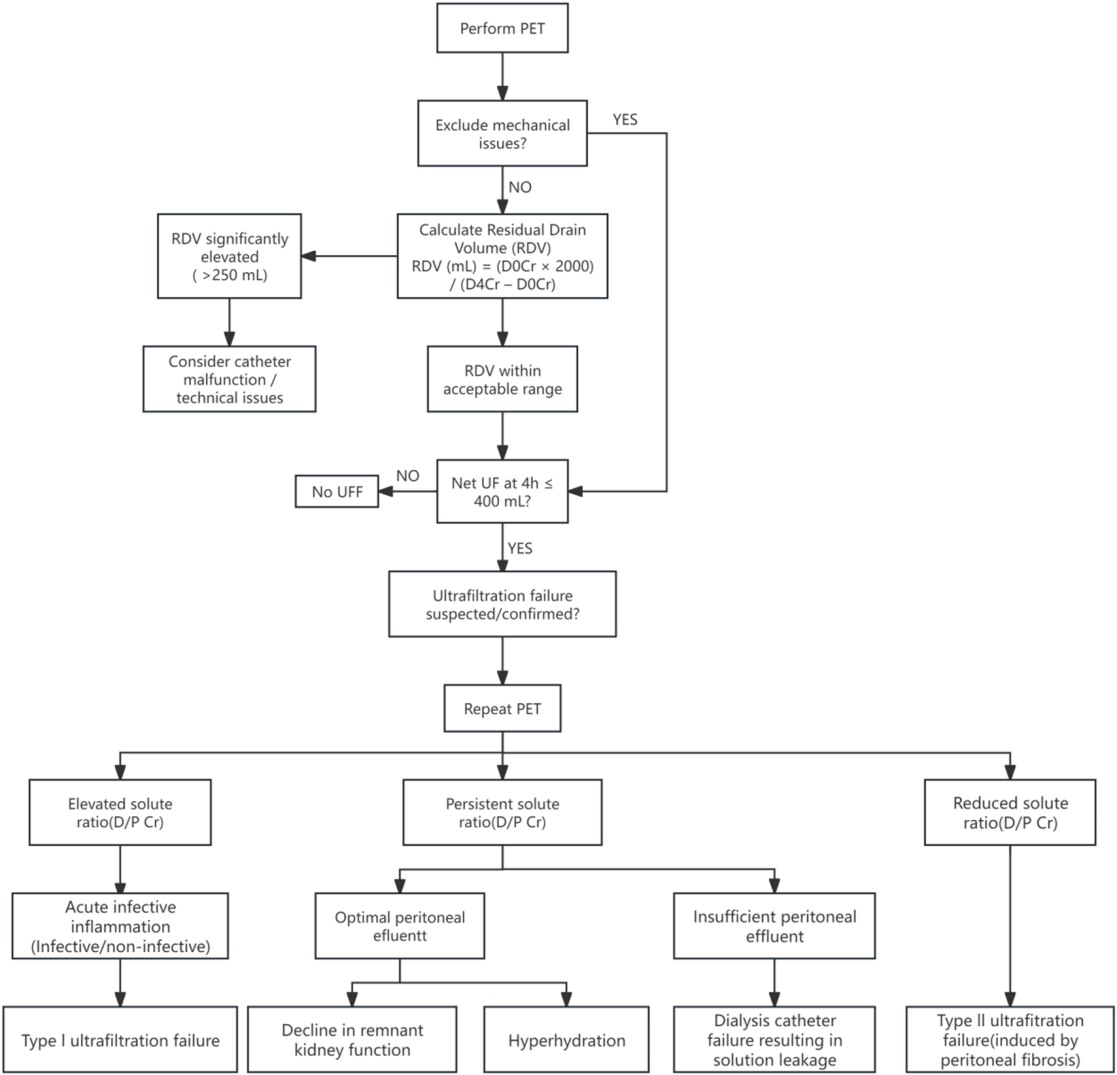
Diagnosis of causes of UUF. D0cr = dialysate creatinine concentration at 0 h; D4cr = dialysate creatinine concentration at 4 h.

A modified PET with a UF of 400 mL or less at 4 h indicated UFF. To exclude false positives due to mechanical issues such as catheter malfunction or incomplete drainage, the residual volume at the start of the PET was assessed. This was objectively estimated using the ‘0-hour’ dialysate sample. The residual drain volume (RDV) was calculated as: RDV (mL) = (D0Cr × D0Vol) / (D4Cr – D0Cr), where D0Cr and D4Cr are the creatinine concentrations (mg/dL) in the dialysate at 0 and 4 h, respectively, and D0Vol is the instilled volume (typically 2000 mL). A significantly elevated RDV suggests incomplete drainage and may confound the UF measurement, prompting re-evaluation of catheter function before attributing UFF to membrane dysfunction.Other reversible causes, including significant loss of residual renal function or excessive fluid intake, were also clinically evaluated and ruled out. The diagnostic utility of PET in identifying the causes of UUF is illustrated in the algorithm (Fig. 1).Elevated rates of small solute clearance (D/PCr) based on the repeated standard PET were observed for type 1 UFF. In patients with type 2 failure, the D/P ratio and sodium sieving reduced. Patients with type 3 failure were defined as having a decreased OCG, while the D/P ratio was persistent and sodium sieving was reduced. Type 4 UFF was defined as a normal D/PCr, and this diagnosis was made by exclusion.

### Calculations

1. We assessed traditional peritoneal transport parameters, including D/PCr, D4/D0 glucose, and UF, at 4 h and 60 min.
2. Sodium sieving was calculated as ∆DNa60 and ∆D/PNa60^8^:
3. ∆DNa60 (mmol/l) = Na dialysate (mmol/l) at the start of the Mini-PET—Na dialysate (mmol/l) at 60 min.
4. ∆D/PNa60 = D/PNa at the start of Mini-PET—D/PNa at 60 min.
5. FWT was determined using the following formula^9^:
6. FWT (mL) = total ultrafiltration (UF) volume at 60 min (mL) *−* UFSP at 60 min (mL).
7. UFSP was calculated as:
8. UFSP (mL) = [sodium removal (mmol) *×* 1000]/Psodium.
9. Sodium removal^10^= [Dialysate V in 1 h (L) *×* Dialysate sodium at 1 h (mmol/L)] *−* [Dialysate V instilled (L) *×* Dialysate sodium at 0 min (mmol/L)]

All variables are expressed as the means ± SD. Statistical analysis was conducted using a paired t test to assess differences between all measured parameters. Continuous variables are presented as mean ± standard deviation. Due to limited sample sizes, formal statistical comparisons were not performed among the four individual UFF subtypes (Table 3) or among the four peritoneal transport groups (Table 4). Based on pathophysiological rationale, patients were categorized into two broader UFF groups (types 1 + 2 vs. types 3 + 4), with between-group differences assessed using the Mann-Whitney U test. This categorization was designed to test the hypothesis that icodextrin efficacy differs between UFF types with structurally preserved versus impaired small-pore function—the primary pathway for its colloid osmotic action. The grouping is based on the principle that types 1 and 2 UFF primarily involve alterations in transport characteristics (fast or slow solute transport, aquaporin function) with a largely intact effective peritoneal surface area (EPSA). In contrast, types 3 and 4 are characterized by a reduced EPSA due to fibrosis or marked lymphatic absorption, which diminishes the capacity for small-pore transport.The Shapiro–Wilk test confirmed that our data were normally distributed; therefore, Pearson’s correlation analysis was used.We performed a correlation analysis between sodium sieving (ΔDNa60) and OCG.Moreover, we conducted a correlation study between the UF of icodextrin and sodium sieving (∆DNa60, ∆D/PNa60) and OCG. Further correlations were analyzed between the UF of icodextrin and the UFSP and FWT during the Mini-PET and Double-Mini PET.

To address the potential confounding effect of prescription heterogeneity, we performed sensitivity analyses. First, we stratified the cohort by dialysis modality and by the use of high-glucose dialysate (4.25% vs. ≤2.5%) to assess the consistency of icodextrin’s effect on ultrafiltration across these subgroups. Second, we analyzed whether the response to icodextrin varied by peritoneal transport type (e.g., high vs. low transporters) by testing for interaction effects in regression models.

## Results

We included 19 patients with UFF (male to female 11/8). One patient had LT, 7 had LAT, 7 had HAT, and 4 patients had HT according to Twardowski’s PET classification (Table 1). Among them, 6 patients had type 1 UFF, 8 had type 2, 3 had type 3, and 2 had type 4 UFF. All patients with UFF were anuric. In the UFF group, fifteen different dialysis fluid regimens with one night exchange of 7.5% icodextrin and three daily exchanges of 2.27% glucose or 3.86% glucose were investigated for each patient. The four remaining patients received automated peritoneal dialysis (APD) using a HomeChoice cycler (Baxter, McGaw Park, IL, USA). They utilized a 2.27% glucose peritoneal dialysis solution (Dianeal) for 8 h overnight and one 10-hour exchange of 7.5% icodextrin solution (Extraneal) during the day.

**Fig. 2:**
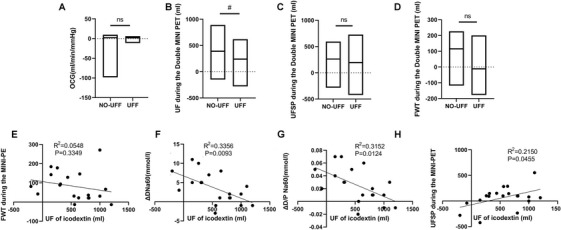
Assessment of icodextrin dialysate and double mini-PET parameters in patients with and without ultrafiltration failure. (A) Comparison of OCG changes between patients with UFF and without UFF during the double Mini-PET. (B) Comparison of UF between patients with UFF and without UFF during the double Mini-PET. (C) Comparison of UFSP between patients with UFF and without UFF during the double Mini-PET. (D) Comparison of FWT between patients with UFF and without UFF during the double Mini-PET. (E) Correlation between UF of icodextrin and FWT measured by Mini-PET. (F) Correlation between UF of icodextrin and ∆DNa60. (G) Correlation between UF of icodextrin and ∆D/PNa60. (H) Correlation between UF of icodextrin and UFSP measured by Mini-PET. UFF: ultrafiltration failure. OCG: osmotic conductance to glucose; UF: ultrafiltration; UFSP: UF through small pores; FWT: free water transport. #: *P* < 0.05 was considered as statistically significant. ns: not significant.

Baseline peritoneal transport characteristics for the UFF group were D/PCr 0.71 ± 0.14 and D4/D0 glucose 0.46 ± 0.16, as shown in Table 2. During the Mini-PET at one hour, the following values were observed: ∆D/PNa60 was 0.02 ± 0.03, FWT was 82.13 ± 75.99 mL, while UFSP was 64.73 ± 214.48 mL, and Net UF was 169.47 ± 218.24 mL.The descriptive values for UF produced by icodextrin, ∆D/PNa60, and FWT across the four UFF types are summarized in Table 3. Numerically, the UF with icodextrin was greatest in the type 1 UFF group, followed by type 2, and was lower in type 3 and type 4 groups. Similarly, FWT appeared lowest in the type 1 UFF group and the highest in type 3. Other parameters, including D/PCr, sodium removal, OCG, UFSP, and net UF from Mini-PET, Double Mini-PET, or modified PET, showed variation among the groups as described in Table 3. When patients were categorized by peritoneal membrane transport type (HT, HAT, LAT, LT), descriptive variations were also observed (Table 4). Notably, the net UF with icodextrin was numerically highest in the HT group.To explore the hypothesis that icodextrin efficacy differs between UFF types with preserved versus impaired small-pore function, patients were grouped into types 1 + 2 (*n* = 14) and types 3 + 4 (*n* = 5). The net UF with icodextrin was numerically higher in the types 1 + 2 group (588.33 ± 103.0 mL) compared to types 3 + 4 group (403.75 ± 202.52 mL), but this difference did not reach statistical significance (*p* = 0.582, Mann-Whitney U test).There were no differences in D/PCr, D4/D0 glucose, UFSP, OCG, or UF of icodextrin between patients with UFF and without UFF (Table 2). Compared with patients without UFF, those with UFF presented a significantly lower FWT during Mini-PET (*p* = 0.038). Patients with UFF demonstrated a significant decrease in net UF during modified PET (*p* = 0.001) but also a significant decrease in net UF during Mini PET (*p* = 0.047) and Double Mini PET (*p* = 0.015) (Table 2). But there were no differences in OCG, UFSP and FWT between patients with UFF and without UFF during the double Mini-PET (Fig. 2A-D).

**Table 2:** Peritoneal ransport characteristic from PET (after 4-h dwell time), miniPET (after 1 h of dwell time), double miniPET (after 1 h of dwell time) and modified PET (after 4 h of dwell time), sodium removal, D/D0 and D/PCre for 19 patients (mean ± SD).

Parametersa	UFF patients *n* = 19	NUFF patients *n* = 24	Value
PET D/P creatinine D4/D0 glucose	0.71 ± 0.14 0.46 ± 0.16	0.65 ± 0.13 0.48 ± 0.15	0.131 0.727
MiniPET Net ultrafiltration (mL) Ultrafiltration through small pores (mL) Free water transport (mL) ∆D_Na60_ (mmol/l) ∆D/P_Na60_ Double miniPET OCG (uL/min/mmHg) Net ultrafiltration (mL) Modified PET Net ultrafiltration (mL) Icodextrin Net ultrafiltration (mL)	169.47 ± 218.24 64.73 ± 214.48 82.13 ± 75.99 3.53 ± 3.98 0.02 ± 0.03 1.33 ± 4.07 215.26 ± 179.49 243.79 ± 257.25 549.47 ± 396.51	327.5 ± 274.36 185.68 ± 243.69 129.74 ± 69.58 6.67 ± 3.95 0.05 ± 0.03 -2.81 ± 22.53 378.75 ± 230.36 508.54 ± 214.65 544.08 ± 318.46	0.047* 0.096 0.038* 0.014* 0.006* 0.435 0.015* 0.001* 0.961

PET: peritoneal equilibrium test. MiniPET: net ultrafiltration (netUF60) defined as the difference between the weight of the effluent (Vend) and the weight of the infused fluid (V0). D/P creatinine: dialysate-to-plasma ratio of creatinine. D4/D0 glucose: the ratio between the 4- and 0-h dialysate glucose concentrations. D/ P sodium: dialysate to plasma sodium concentration (D/PNa) at the end (D/PNa60) of miniPET. Dip D/P sodium: the sodium dip DipNa60 = D/PNa0 —D/PNa60. OCG: osmotic conductance to glucose. *: *P* < 0.05 was considered as statistically significant.

**Table 3:** Descriptive data on peritoneal solute transport characteristics, sodium removal, and net ultrafiltration obtained from PET, miniPET, double miniPET and modified PET, stratified by type of UFF.

Type of ultrafiltration failure (*n* = 19)	D/*P* creatinine	Ultrafiltration through small pores (mL)	Free water transport (mL)	∆D_Na60_ (mmol/l)	∆D/*P*_Na60_	Sodium removal	OCG (uL/min/mmHg)	MiniPET Net ultrafiltration (mL)	Double MiniPET Net ultrafiltration (mL)	Modified PET Net ultrafiltration (mL)	Icodextrin Net ultrafiltration (mL)
Type 1(*n* = 6) Median (range) Type2(*n* = 8) Median (range) Type3(*n* = 3) Median (range) Type 4(*n* = 2) Median (range)	0.83 ± 0.37 0.84(0.78–0.87) 0.66 ± 0.15 0.66(0.46–0.97) 0.65 ± 0.09 0.64(0.57–0.74) 0.62 ± 0.04 0.62(0.59–0.64)	59.55 ± 174.81 77.5(-236.22-288.13) 166.96 ± 179.32 100.41(-42.25-553.18) -109.48 ± 273.10 15.49(-422.7-78.78) -67.36 ± 302.14 -67.36(-281-146.29)	48.79 ± 51.02 33.46(-13.13-128.92) 61.79 ± 66.45 36.65(-12.91-178.13) 199.48 ± 65.55 184.51(142.7-271.22) 87.55 ± 8.72 87.55(81.38–93.71)	2.67 ± 2.88 2(-1-7) 2.38 ± 5.29 2.33(-7-10) 3 ± 9.17 5(-7-11) 6.5 ± 2.12 6.5(5–8)	0.02 ± 0.02 0.02(-0.02-0.05) 0.03 ± 0.02 0.02(0-0.07) 0.07 ± 0.04 0.07(0.04–0.11) 0.04 ± 0.01 0.04(0.03–0.05)	8.5 ± 22.97 10.76(-30-38.61) 18.32 ± 27.23 14.01(-18.3-71.36) 19.27 ± 15.9 21.95(2.2-33.66) -10.16 ± 43.33 -10.16(-40.8-20.48)	-0.14 ± 5.67 1.19(-11.31-4.61) 2.67 ± 1.97 3.09(-1.25-5.76) -1.28 ± 4.3 0.96(-6.24-1.44) 4.28 ± 2.65 4.28(2.4–6.15)	108.33 ± 189.68 115(-200-310) 190 ± 236.7 220(-180-620) 336.67 ± 130.51 350(200–460) 20 ± 311.13 20(-200-240)	240 ± 124.42 213.33(120–450) 262.5 ± 175.64 275(70–620) 6.67 ± 253.25 100(-280-200) 265 ± 35.36 265(240–290)	178.67 ± 173.86 245(-100-342) 246.25 ± 281.68 250(-200-770) 330 ± 147.31 250(240–500) 300 ± 147 300(-140-740)	729.17 ± 338.65 715(305–1200) 581.25 ± 361.72 605(-80-1100) 153.33 ± 5.77 153.33(150–160) 57.5 ± 364.16 57.5(-200-315)
Total Median (range)	0.74 ± 0.12 0.74(0.46–0.97)	64.73 ± 214.86 96.43(-422.7-553.18)	82.13 ± 75.99 66.82(-13.14-271.22)	3 ± 4.93 3(-7-11)	0.03 ± 0.03 0.03(-0.01-0.11)	12.37 ± 25.54 13.87(-40.8-71.36)	1.33 ± 4.07 2.11(-11.31-6.15)	169.47 ± 218.24 200(-200-620)	215.26 ± 179.49 240(-280-620)	243.79 ± 257.25 250(-200-770)	505.26 ± 390.27 550(-200-1200)

TCUF: transcapillary ultrafiltration. PET: peritoneal equilibrium test. MiniPET: net ultrafiltration (netUF60) defined as the difference between the weight of the effluent (Vend) and the weight of the infused fluid (V0). UFSP: Ultrafiltration through small pores. D/ P sodium: dialysate to plasma sodium concentration (D/PNa) at the end (D/PNa60) of miniPET. Dip D/P sodium: the sodium dip DipNa60 = D/PNa0 —D/PNa60. Ultrafiltration through small pores UFSP60 = RemNa/CBNa, where sodium removal RemNa ¼ Vend CNaend- V0 CNa0; free water transport FWT = netUF60—UFSP60; free water fraction FWF = FWT/netUF60. OCG: osmotic conductance to glucose. *: *P* < 0.05 was considered as statistically significant.Total: the overall group.Data are presented as mean ± standard deviation. No inferential statistics are reported for comparisons among the four subtypes due to small sample sizes.

**Table 4:** Descriptive data on peritoneal solute transport parameters obtained from, PET, miniPET, double miniPET and modified PET, stratified by peritoneal transport type.

Type of PET (*n* = 19)	D/*P* creatinine	Ultrafiltration through small pores (mL)	Free water transport (mL)	∆D_Na60_ (mmol/l)	∆D/*P*_Na60_	Sodium removal	OCG (uL/min/mmHg)	MiniPET Net ultrafiltration (mL)	Double MiniPET Net ultrafiltration (mL)	Modified PET Net ultrafiltration (mL)	Icodextrin Net ultrafiltration (mL)
HT(*n* = 4) Median (range) HAT(*n* = 7) Median (range) LAT(*n* = 7) Median (range) LT(*n* = 1)	0.85 ± 0.02 0.86(0.83–0.87) 0.78 ± 0.09 0.76(0.73–0.97) 0.59 ± 0.04 0.58(0.54–0.64) 0.46	28.59 ± 215.67 31.22(-236.22-288.13) 102.1 ± 283.77 98.95(-422.7-533.18) 16.84 ± 145.33 78.78(-281-146.29) 282.91	18.91 ± 22.17 26.29(-13.14-36.22) 89.33 ± 51.97 88.14(21.29–146.4) 124.64 ± 89.66 93.71(21.29-271.22) -12.91	1 ± 1.41 1.33(-1-2) 4.14 ± 5.84 5(-7-11) 3.57 ± 5.53 4.33(-7-10) -1	0.01 ± 0.01 0.01(-0.01-0.02) 0.04 ± 0.02 0.04(0.01–0.07) 0.05 ± 0.03 0.04(0.01–0.11) 0	4.29 ± 28.24 4.28(-30-38.61) 22.04 ± 26.94 19.96(-18.3-71.36) 3.67 ± 21.99 13.72(-40.8-21.95) 37.91	-1.56 ± 6.57 1.19(-11.31-2.69) 1.88 ± 3.94 3.17(-6.24-5.76) 2.12 ± 2.25 2.11(-1.25-6.15) 3.46	47.5 ± 208.55 40(-200-310) 252.86 ± 255 280(-180-620) 141.43 ± 188.36 200(-200-350) 270	245 ± 155.03 205(120–450) 222.86 ± 271.58 280(-280-620 182.86 ± 92.14 200(70–290) 270	172.5 ± 187.15 245(-100-300) 241.71 ± 296.05 240(-200-770) 267.14 ± 293.7 250(-140-740) 380	907.5 ± 246.76 900(630–1200) 570.71 ± 317.31 550(150–1100) 202.14 ± 324.4 160(-200-810) 560
Total Median (range)	0.71 ± 0.14 0.74(0.46–0.97)^&^	64.73 ± 214.48 96.43(-422.7-553.18)^&^	82.13 ± 75.99 66.82(-13.14-271.22)^&^	3 ± 4.93 3(-7-11)^&^	0.03 ± 0.03 0.03(-0.01-0.11)^&^	12.37 ± 25.54 13.87(-40.8-71.36)^&^	1.33 ± 4.07 2.11(-11.31-6.15)^&^	169.47 ± 218.24 200(-200-620)^&^	215.26 ± 179.49 240(-280-620)^&^	243.79 ± 257.25 250(-200-770)^&^	505.26 ± 390.27 550(-200-1200)

PET: peritoneal equilibrium test. HT: hight transporters. HAT: high average transporter. LAT: low-average transporter. MiniPET: net ultrafiltration (netUF60) defined as the difference between the weight of the effluent (Vend) and the weight of the infused fluid (V0). D/ P sodium: dialysate to plasma sodium concentration (D/PNa) at the end (D/PNa60) of miniPET. Dip D/P sodium: the sodium dip DipNa60 = D/PNa0 —D/PNa60. OCG: osmotic conductance to glucose. *: *P* < 0.05 was considered as statistically significant.Total: the overall group.Data are presented as mean ± standard deviation. No inferential statistics are reported for comparisons among the four subtypes due to small sample sizes.

The results of sensitivity analyses indicated that the ultrafiltration improvement associated with icodextrin was consistent across different dialysis modalities (CAPD vs. APD/IPD) and glucose concentration subgroups (4.25% vs. ≤2.5%). Furthermore, no significant interaction was found between peritoneal transport type and icodextrin efficacy, suggesting that its effect did not differ substantially by transport status (*p* > 0.05).

No significant correlation was observed between sodium sieving (ΔDNa60) and OCG (*P* > 0.05). Negative correlation was found between net UF of icodextrin and ∆D/PNa60 (R^2^ = 0.3152, *P* = 0.0124) and ∆DNa60 (R^2^ = 0.3356, *P* = 0.0093) (Fig. 2E-G). Further correlations were analyzed to evaluate the UF of icodextrin and the UFSP and FWT during Mini-PET. As shown in Fig. 2H, the net UF of icodextrin was positively correlated with the UFSP of Mini-PET (R^2^= 0.215, *p* = 0.0455).

## Discussion

UFF is a serious complication of PD, often necessitating discontinuation of treatment. UFF can be categorized into early UFF (occurring within 2 years of PD initiation) and late UFF. Early UFF occurs in approximately 4% of newly diagnosed PD patients and typically does not present significant clinical issues^11^. In contrast, patients treated for over 5 years tend to develop more severe UFF^12^. In this study, the PD duration in the UFF group was significantly longer than in the non-UFF group, with a mean of 76.21 ± 40.45 months (Table 1), indicating that most UFF cases were late UFF. Most patients undergoing long-term PD tend to experience a decrease in sodium sieving over time. This reduction is a strong and independent predictor of encapsulating peritoneal sclerosis (EPS)^13^. In our study, the mean ∆DNa60 in the UFF group was markedly lower than the normal level (3.53 ± 3.98 vs. 9 mmol/l), suggesting an increased risk of EPS in these patients. Long-term UFF may impair both FWT and UFSP. Calculations in patients indicate that UFSP contributes to 60% of UF, whereas FWT accounts for 40%^11^. Patients with EPS exhibit significantly reduced FWT, likely due to disruption of the crystalloid osmotic gradient by interstitial collagen-1. This mechanism might also be relevant for other patients experiencing a decreased FWT related to the loss of EPSA due to sclerosis or adhesion^14^. In this study, FWT was lower in the UFF group, which might be related to peritoneal membrane sclerosis or adhesion.

Icodextrin is a high-molecular-weight glucose polymer derived from starch, primarily connected by α-1,4 glycosidic bonds^15^. The strong correlation between the ultrafiltration volume and icodextrin may be attributed to icodextrin’s ability to enhance UFSP^16^. We found a positive correlation between net UF with icodextrin UFSP during Mini-PET (*p* = 0.042) in the UFF group. However, net UF of icodextrin was not negatively correlated with FWT during Mini-PET (*p* = 0.335). This result aligns with previous studies^6^. It is important to emphasize that the observed lower sodium sieving in these patients reflects impaired FWT through aquaporins. In contrast, the ultrafiltration achieved with icodextrin depends primarily on UFSP rather than aquaporin-mediated pathways.With icodextrin, water is transported through small pores without sodium sieving, suggesting that water does not pass through ultrasmall pores (aquaporins)^17^. The degree of sodium sieving reflects AQP function. Furthermore, sodium sieving serves as an indirect indicator of FWT^18^. Some researchers have proposed correcting peritoneal sodium diffusion using the mass transport area coefficient (MTAC)^19^. Even after correcting for MTAC, no significant correlation was observed between the net UF of icodextrin and FWT, a result that will be confirmed by future studies.

FWT can be readily evaluated using the mini-PET, which measures sodium sieving through a reduction in the dialysate sodium concentration (∆DNa60) or a decrease in the ratio of the dialysate to plasma sodium concentration (∆D/PNa60)^8^. This study revealed a significant correlation between the net UF of icodextrin and ∆D/PNa60 (*P* = 0.0124), contrasting previous studies. Rodríguez Carmona et al. reported that while icodextrin enhanced sodium removal during dialysis in CAPD and APD patients in univariable analyses, it had no significant effect on sodium removal when controlling for UF^20^. This suggests that icodextrin may enhance sodium removal primarily by increasing UF rather than having an independent effect on sodium clearance. This finding indicates that icodextrin solutions can enhance sodium removal primarily through their ability to increase the UF. Our study found no significant correlation (*P* > 0.05) between net UF of icodextrin and sodium removal. However, significant correlations were observed between net UF of icodextrin and ∆D/PNa60 (*P* = 0.0124) and ∆DNa60 (*P* = 0.0093). Nevertheless, a significant correlation persisted (*r* = 0.57, *P* = 0.005) between net UF of icodextrin and ∆D/PNa60 but not ∆DNa60 after controlling for UF. This is because ∆D/PNa60 depends on fluctuations in the plasma sodium concentration over time and the method employed to measure plasma sodium levels. Furthermore, as estimated by ∆DNa60, sodium sieving exhibited the highest sensitivity and specificity among the parameters evaluated. Therefore, ∆DNa60 is the most straightforward and reliable tool for assessing FWT^21^.

∆DNa60 demonstrates a consistent and significant decrease over time and is the only parameter independently predicting the risk of developing UFF in patients undergoing PD. The decline in sodium sieving is a strong and independent predictor of EPS^8^. Morelle et al. reported that this decline occurred significantly faster in patients with EPS. Their findings revealed that a ∆DNa60 < 5 mmol/L or a sodium sieving ratio below 0.03 was considered a high risk for developing EPS^22^. In our study, all UFF patients exhibited ∆DNa60 below 5 mmol/L, a threshold associated with high risk for EPS in the literature^22^.Descriptively, the mean ∆D/PNa60 appeared lower in the type 1 and 2 UFF groups compared to other types (Table 3). This pattern suggests that impaired FWT, as reflected by low sodium sieving, might be more pronouned in these UFF subtype, which could theoretically be associated with is an increased risk of EPS. The FWT influenced by sodium sieving can be impacted by functional AQP1 expression and factors obstructing the membrane, such as significant peritoneal fibrosis^23^. Because FWT was numerically lower in the type 1 UFF group, most UFF 1 patients had EPS.Morelle et al. demonstrated that the vascular density of water channels remained unchanged in patients with EPS, highlighting that excessive peritoneal fibrosis is the primary barrier to fluid flow in these individuals. This finding may explain why patients with type 1 and 2 UFF in our study experienced numerically higher UF of icodextrin^22^. Therefore, based on our findings, we hypothesize that a 7.5% icodextrin solution could be a potentially effctive fluid management option for PD patients with type 1 and 2 UFF, warranting further investigation. This study represents the first report verifying whether a 7.5% icodextrin solution is suitable for all PD patients with UFF.

The OCG has rarely been evaluated in patients undergoing PD.In our cohort, the directly measured OCG did not show a significant correlation with the simpler parameter ΔDNa60, possibly due to sample size limitations and the methodological challenges associated with the Double Mini-PET. Specifically, OCG calculation is methodologically vulnerable to drained volume measurement errors, especially in UFF patients and substantial residual volume^24^.Nonetheless, the reduction in ΔDNa60 itself served as a useful functional marker in distinguishing UFF patients.A decrease in OCG may be linked to impaired AQP function^25^. Stelin and Rippe reported a mean OCG of 3.54 ± 2.90 ml/min/mm Hg in a cohort of 12 PD patients without UFF^26^. This aligns closely with the value observed in our patients without UFF, which was 3.04 ml/min/mm Hg. Among all UFF patients, we observed a slight reduction in OCG (1.33 ml/min/mm Hg). A trend toward further reduction eas noted in type 1 and 3 UFF patients, although no formal statistical comparions were made due to sample size limitations. Pereira et al. reported that in patients with a severely decreased OCG, increasing the glucose concentration in the PD solutions or reducing the dwell time was not beneficial. Instead, prescribing icodextrin solutions is more appropriate^5^. In our study, the HTs and type 1 UFF patients with low OCG concentrations demonstrated the best net UF of icodextrin. Because HT patients exhibit greater vascularization of the peritoneal membrane, they have a higher number of small pores. This condition allows icodextrin to induce colloid osmosis more effectively, resulting in improved ultrafiltration in these patients^27^.

In contrast, the mean net UF of icodextrin was numerically reduced in patients with type 3 UFF. In addition to the concentration gradient between plasma and intra-abdominal dialysate, the density of small pores—reflecting the number of perfused peritoneal microvessels—is crucial in overall diffusion. This parameter is also referred to as the EPSA. A larger EPSA is associated with higher ultrafiltration rates of icodextrin^28^. The D/PCr ratio is frequently utilized as a functional parameter of EPSA. Because D/PCr was numerically lower in the UFF 3 group than in the UFF 1 and 2 groups, the net UF of icodextrin appeared lowed in patients with type 3 UFF due to the small EPSA. Our study validated that a 7.5% icodextrin solution may be a viable option for PD patients with type 1 UFF, its utility in type 3 UFF could be limited, warranting further investigation in larger cohorts.

Icodextrin is a large molecule, resulting in minimal absorption from the peritoneal cavity. Only 10%–20% of the infused macromolecules are absorbed by the lymphatic vessels from the peritoneal cavity, which helps sustain a continuous oncotic pressure gradient^29^. Consequently, lymphatic absorption from the peritoneal cavity is a critical factor influencing ultrafiltration volume during PD, particularly for short-term patients. Our study revealed that icodextrin yielded numerically the poorest UF results in the type 4 UFF group, further supporting this conclusion. This finding also indicates that icodextrin is not suitable for PD patients with type 4 UFF.

Contrary to our initial pathophysiological hypothesis, the difference in net ultrafiltration with icodextrin between the combined group of UFF types 1 + 2 and types 3 + 4 did not reach statistical significance (*p* = 0.582). The reason is likely due to limited sample size, particularly in the latter group (*n* = 5), resulting in low statistical power. The observed numerical trend favoring types 1 + 2 requires validation in larger studies. Potential heterogeneity within UFF subtypes and the complex mechanism of icodextrin action—which may be less effective against the distinct pathophysiology of types 3 and 4 —could also contribute to the findings. Therefore, while our descriptive data suggest that icodextrin may be more suitable for types 1–2 than for 3–4, this hypothesis must be confirmed in larger cohorts using continuous functional parameters rather than categorical classification**s.**

We acknowledge that our UFF cohort included a wide variety of peritoneal dialysis prescriptions, Patients were treated with CAPD, APD, or IPD, and the dialysate glucose concentrations ranged from 1.25% to 4.25%, with various combinations and exchange numbers. This diversity reflects real-world clinical practice, where prescriptions are individualized based on patient needs and peritoneal membrane characteristics.The observed association between icodextrin and improved ultrafiltration appeared robust in sensitivity analyses. To address potential confounding from prescription heterogeneity, we stratified analyses by dialysis modality (CAPD vs. APD/IPD) and by the use of high-glucose dialysate (4.25% vs. ≤2.5%). The beneficial effect of icodextrin was consistent across these subgroups. Furthermore, we found no significant interaction between peritoneal transport type and icodextrin efficacy, suggesting that its effect is not substantially modified by underlying membrane transport characteristics. These analyses support the generalizability of our primary findings across clinically relevant patient subgroups.While we cannot fully exclude residual confounding due to individualized prescriptions, these analyses suggest that the observed benefit of icodextrin is robust across a range of real-world regimens.

This study had several shortcomings. First, the sample size was relatively small,, especially within individual UFF subtypes, which limits statistical power and increases susceptibility to outliers. The high variability and occurrence of physiologically implausible values (e.g.negative OCG) in these small subgroups warrant cautious interpretation and likely reflect inherent patient heterogeneity, very low ultrafiltration volumes, and the technical challenges of precise volume measurement in UFF. These methodological constraints, well-documented in the literature^24,30^ underscore the need for careful interpretation of quantitative transport parameters in severe UFF.Despite the limited number of patients with UFF, we can draw some intriguing insights into the mechanisms underlying UFF. This confirms that the UF obtained with icodextrin is more effective in forecasting the UFF pattern. The UFF 1 and 2 patients had numerically higher net UF of icodextrin. Second, we did not calculate the effective lymphatic absorption rate and could not provide more information for type 4 UFF. Third, the typical or abnormal values of the standard parameters of the OCG are not yet clear.Additionally, due to its retrospective design, we lacked direct measurement of sodium clearance during the long dwell with icodextrin, which is a key clinical efficacy endpoint for fluid and sodium management. Our finding of a positive correlation between icodextrin net UF and UFSP (but not with FWT) is consistent with the established mechanism that icodextrin enhances sodium removal primarily by increasing convective transport through small pores. Future prospective studies should include this direct measure of sodium removal to complement the pathophysiological insights provided by parameters like FWT and UFSP, and to better quantify the clinical benefit of icodextrin.

In summary, this exploratory study provides preliminary insights into the differential effectiveness of a 7.5% icodextrin solution across UFF subtypes, with a focus on distinct water transport pathways. Our findings demonstrate that icodextrin may be suitable for patients with type 1 and 2 UFF, but appears perhaps less effective in patients with type 3 and type 4 UFF, where small-pore transport is impaired.Furthermore, we observed a positive correlation between the net UF of icodextrin and the UFSP of Mini-PET. In the future, the UF of icodextrin can be predicted based on UFSP levels. The limited sample size also underscores the need for further research involving a larger patient population.

## Data Availability

The datasets used and/or analysed during the current study available from the corresponding author on reasonable request.
